# *Bacillus subtilis* RecA, DisA, and RadA/Sms Interplay Prevents Replication Stress by Regulating Fork Remodeling

**DOI:** 10.3389/fmicb.2021.766897

**Published:** 2021-11-22

**Authors:** Rubén Torres, Juan C. Alonso

**Affiliations:** Department of Microbial Biotechnology, Centro Nacional de Biotecnología, CNB-CSIC, Madrid, Spain

**Keywords:** DNA repair, c-di-AMP, fork stalling, fork reversal, template switching, Holliday junction

## Abstract

Reviving *Bacillus subtilis* spores require the recombinase RecA, the DNA damage checkpoint sensor DisA, and the DNA helicase RadA/Sms to prevent a DNA replication stress. When a replication fork stalls at a template lesion, RecA filaments onto the lesion-containing gap and the fork is remodeled (fork reversal). RecA bound to single-strand DNA (ssDNA) interacts with and recruits DisA and RadA/Sms on the branched DNA intermediates (stalled or reversed forks), but DisA and RadA/Sms limit RecA activities and DisA suppresses its c-di-AMP synthesis. We show that RecA, acting as an accessory protein, activates RadA/Sms to unwind the nascent lagging-strand of the branched intermediates rather than to branch migrate them. DisA limits the ssDNA-dependent ATPase activity of RadA/Sms C13A, and inhibits the helicase activity of RadA/Sms by a protein-protein interaction. Finally, RadA/Sms inhibits DisA-mediated c-di-AMP synthesis and indirectly inhibits cell proliferation, but RecA counters this negative effect. We propose that the interactions among DisA, RecA and RadA/Sms, which are mutually exclusive, contribute to generate the substrate for replication restart, regulate the c-di-AMP pool and limit fork restoration in order to maintain cell survival.

## Introduction

Complete, accurate and timely DNA replication is essential to maintain genome integrity and cell proliferation. However, replicative DNA polymerases, which are generally poor at synthesizing past lesions, are frequently hindered by obstacles, and the replication fork stalls (Cox et al., 2000; Mirkin and Mirkin, 2007). In most bacteria, replication of DNA containing damaged template bases or DNA distortions can lead to fork reversal (also named fork regression), i.e., the coordinated annealing of the two nascent strands, leading to a structure resembling a Holliday junction (HJ) (Atkinson and McGlynn, 2009; Marians, 2018). This fork remodeling mechanism has emerged as a global and genetically controlled response to aid repair or bypass of DNA damage upon replication stress during the early stage of *Bacillus subtilis* spore revival (Vlasic et al., 2014; Raguse et al., 2017) as well as in mammalian cells (Branzei and Foiani, 2010; Neelsen and Lopes, 2015; Quinet et al., 2017; Berti et al., 2020). By contrast, in *Escherichia coli*, when replication forks encounter template lesions they are simply skipped, but replication-transcription conflicts mostly trigger fork reversal (Marians, 2018; Wong et al., 2021).

When the DNA of an inert mature haploid spore is damaged by exposing to ionizing radiation, and then the spores are synchronously revived under unperturbed conditions, fork processing or breakage should be lethal because end resection functions are transiently absent and only one copy of the genome is available, and thus homologous recombination cannot operate (Vlasic et al., 2014; Raguse et al., 2017). Indeed, in the absence of both end resection pathways (i.e., in the Δ*recJ* Δ*addAB* strain), which drive the first step of homologous recombination, haploid reviving spores remain recombination proficient and are as capable of repairing the pre-existing ionizing radiation-induced damage as the wild type (*wt*) control (Vlasic et al., 2014). In reviving spores, the ionizing radiation-induced broken ends are simply reconnected by Ku and LigD *via* non-homologous end joining, and the nicks repaired *via* LigD-dependent pathways during the ripening period of spore revival (Wang et al., 2006; de Ory et al., 2016). Then, at the early stage of spore outgrowth and concomitant with DNA replication, the unrepaired offending lesions stall replication fork progression, with fork reversal/template switching or lesion bypass emerging as a reversible and genetically controlled transaction to maintain genome integrity. Here, the recombinase RecA, its accessory proteins (e.g., RecO, RecR, RecF), the DNA translocases (RecG and RuvAB), the DNA damage checkpoint sensor DisA and the DNA helicase RadA/Sms are required for spore survival (Vlasic et al., 2014; Raguse et al., 2017). Finally, long-range end-resection functions accumulate at a later stage of outgrowth and prior to cell elongation (Nicolas et al., 2012; Sinai et al., 2015). (Unless stated otherwise, indicated genes and products are of *B. subtilis* origin).

Genetic data revealed that inactivation of *disA* renders exponentially growing cells sensitive to bulky and non-bulky lesions that cause replication arrest, but inactivation of *radA* or *recA* renders cells sensitive and extremely sensitive, respectively, to damaging agents that introduce bulky and non-bulky lesions, single strand nicks or DSBs (Gándara et al., 2017; Raguse et al., 2017). It is likely, therefore, that DisA selectively acts at stalled forks, whereas RadA/Sms and RecA have a much broader role in recombinational repair (Gándara et al., 2017). Cytological data revealed that upon exposure to UV, RecA colocalizes with the stalled replisome in up to 85% of exponentially growing cells, as early as 5 min after treatment (Simmons et al., 2007), suggesting that lesion-skipping may not represent the primary pathway to overcome a replicative stress. Here, cells respond to a genotoxic insult by disengaging the replisome, protecting it and exposing the stalled fork for remodeling (Mangiameli et al., 2017). DisA provides a DNA damage checkpoint that delays entry into sporulation until the offending lesion is removed (Bejerano-Sagie et al., 2006). DisA forms a fast-moving focus that pauses in response to DNA damage in sporulating cells (Bejerano-Sagie et al., 2006). DisA pausing requires RecA, but not AddAB and RecJ (Torres et al., 2019a), suggesting that the signal(s) recognized by DisA should be formed when RecA is engaged with branched intermediates [e.g., a stalled (an isomer of a displacement loop, D-loop) or reversed forks (a HJ-like structure)] rather than with DNA ends. In unperturbed exponentially growing *wt* cells, dynamic DisA-YFP foci or RadA/Sms-YFP foci mostly co-localize with the DNA bulk, but in genetic backgrounds that accumulate branched intermediates (e.g., Δ*recG* cells), both proteins paused and transiently co-localized in ∼27% of the cells (Gándara et al., 2017). This is consistent with the observation that RecA, DisA, and RadA/Sms physically interact among them (Torres et al., 2019a,b,c).

**FIGURE 1:**
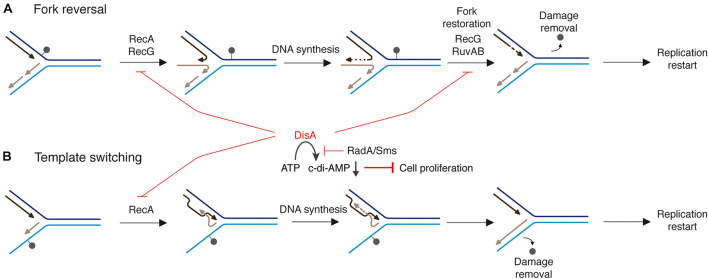
Proposed mechanisms for RecA, DisA, RecG, RuvAB, and RadA/Sms in DNA damage tolerance pathways. When a replication fork encounters template DNA lesions, RecA assembles at the lesion-containing gap. RecA interacts with and loads DisA and RadA/Sms. Two error-free DDT pathways [fork reversal **(A)** and template switching **(B)**] contribute to place the lesion on duplex DNA to permit repair and to generate the substrate for DNA synthesis to resume. **(A)** An unrepaired DNA lesion on the leading strand template (black dot) causes blockage of replication fork movement. A fork remodeler (e.g., RecG) reverses the fork by annealing the two nascent strands, with DisA limiting RecG activities. DNA synthesis of the DNA complementary to the damaged site (denoted by a dotted line) is followed by fork restoration catalyzed by RecG or RuvAB. Then, RadA/Sms with the help of RecA generates the substrate to be recognized by the pre-primosomal protein for replication re-start. **(B)** An unrepaired DNA lesion on the lagging strand template (black dot) causes blockage of replication fork movement. Finally, a substrate to be recognized by the pre-primosomal proteins for replication re-start should be generated.

Taking these data into account, we hypothesized that an interplay between RecA and DisA or RadA/Sms could provide a mechanism to cope with a replicative stress (Figure 1). Previously it has been shown that when replication forks encounter template DNA lesions in the leading- or lagging-strand, RecA filaments in the lesion-containing gap (Figures 1A,B). RecA, assembled at a stalled/reversed fork, interacts with and loads DisA and RadA/Sms onto these branched intermediates, but DisA and RadA/Sms inhibit the ATPase activity of RecA (Torres et al., 2019a,b,c), suggesting that DisA and/or RadA/Sms might regulate the dynamic of a RecA filament (Torres et al., 2019a). The fork is remodeled, perhaps by RecG, with DisA limiting RecG activities (Figure 1A; Torres et al., 2021). If the offending lesion is in the lagging-strand, RecA catalyzes template switching, with DisA limiting RecA activities (Figure 1B). DisA is composed of an N-terminal globular domain with diadenylate cyclase (DAC) activity and a C-terminal RuvA-like HJ DNA-binding domain (Witte et al., 2008). Octameric DisA converts a pair of ATPs into a cyclic 3′, 5′-diadenosine monophosphate (c-di-AMP) molecule, an essential second messenger that regulates a variety of mechanisms in the cell (Stulke and Kruger, 2020). In response to lesions that stall replication, the amount of c-di-AMP drops in *wt* cells to levels comparable to that in the absence of DisA *in vivo* (Gándara and Alonso, 2015), and *in vitro* DisA bound to branched intermediates (a stalled or reversed fork) reduces (Witte et al., 2008; Gándara et al., 2017) and upon interaction with RadA/Sms blocks c-di-AMP synthesis (Torres et al., 2019c). Low c-di-AMP levels increase (p)ppGpp production, which in turn inhibits the DNA primase and indirectly cell proliferation (Figures 1A,B; Wang et al., 2007; Kriel et al., 2012). It is likely that a fail-safe mechanism to coordinate the cell cycle and maintain cell survival when there are obstacles that may hinder the progression of the replication fork is provided by DisA. RadA has four well-conserved motifs: a C4-type zinc-binding in the N-terminal domain, a central canonical RecA-like ATPase domain (H1-H4 motifs), a KNRFG motif, and a P dumbbell-shaped homohexameric domain at the C-terminus (Marie et al., 2017). Upon DNA damage, RadA/Sms, or *Mycobacterium tuberculosis* RadA (RadA*_*Mtu*_*) interacts with and blocks the DAC activity of its cognate DisA (Zhang and He, 2013; Gándara et al., 2017). *In vitro*, RadA/Sms binds ssDNA and branched structures with similar high affinity and unwinds DNA by moving unidirectionally in the 5′→ 3′direction (Marie et al., 2017; Torres et al., 2019b). Upon interacting with RecA, *wt* RadA/Sms unwinds substrates (as 5′fork DNA) that RecA cannot process by itself (Figures 1A,B; Marie et al., 2017; Torres et al., 2019b), but limits RecA activities (Torres et al., 2019b). In contrast, RadA*_*Eco*_* seems to lack any DNA helicase activity (Cooper and Lovett, 2016).

Taking these data into account, we assumed that these proteins contribute to fork remodeling, thereby limiting fork breakage in reviving spores. However, the interplay of RecA, DisA and RadA/Sms during a replicative stress remain elusive. In this work, using biochemical approaches, we have investigated their interplay. We show that: (i) RecA stimulates DisA and RadA/Sms activities, but DisA restrains RadA/Sms and RecA activities and RadA/Sms limits DisA and RecA activities; (ii) RecA bound to stalled or reversed forks facilitates RadA/Sms-mediated reconstitution of the fork to restart replication, but DisA inhibits it; and (iii) RecA reverses the negative effect exerted by RadA/Sms on DisA DAC activity. We propose that fork remodeling is subjected to distinct layers of regulation. RecA interacts with and loads DisA and RadA/Sms at a stalled or reversed fork. DisA bound to these branched intermediates inhibits c-di-AMP synthesis, and RadA/Sms blocks it; with low c-di-AMP levels indirectly halting cell proliferation. Then, DisA limits RecA dynamics, and paused RecA facilitates RadA/Sms unwinding of non-cognate reversed forks, a reaction limited by DisA. Once the lesion is removed, RecA indirectly antagonizes the blockage of cell proliferation by dislodging RadA/Sms, allowing the DAC activity of DisA to be turned on, and reversing the cell proliferation blockage.

## Materials and Methods

### Bacterial Strains and Plasmids

*E. coli* BL21(DE3) [pLysS] cells bearing pCB1020 (*radA*), pCB1037 (*radA* K104A), pCB1035 (*radA* C13A), pCB875 (*disA*), pCB1081 (*disA* ΔC290), and pQE-1 (*pcrA*) genes under the control of a rifampicin-resistant promoter (P_*T*__7_) were used to overproduce RadA/Sms (the slash between RadA and Sms names denotes that it has alternative names, to avoid confusion with RadA of Archaea; the gene is termed *radA*), RadA/Sms K104A, RadA/Sms C13A, DisA, DisA ΔC290, and PcrA proteins, respectively, as described (Gándara et al., 2017; Torres et al., 2019a,b,c; Moreno-Del Alamo et al., 2020). *B. subtilis* BG214 cells bearing pBT61 (*recA*) gene were used to overproduce RecA (Carrasco et al., 2005).

### Enzymes, Reagents, Protein, and DNA Purification, Protein-Protein Interaction

All chemicals used were analytical grade. IPTG (isopropyl-β-D-thiogalactopyranoside) was from Calbiochem (Darmstadt, Germany), DNA polymerases, DNA restriction enzymes, and DNA ligase were from New England Biolabs (Ipswich, MA), and polyethyleneimine, DTT, ATP, and dATP were from Sigma (Seelze, Germany). DEAE, Q- and SP-Sepharose were from GE Healthcare (Marlborough, MA), hydroxyapatite was from Bio-Rad (Hercules, CA), phosphocellulose was from Whatman (Maidstone, Kent, United Kingdom), and the Ni-column was from Qiagen (Hilden, Germany).

The proteins RadA/Sms (49.4 kDa), RadA/Sms K104A (49.4 kDa), RadA/Sms C13A (49.4 kDa), DisA (40.7 kDa), DisA ΔC290 (33.5 kDa), PcrA (83.5 kDa), and RecA (38.0 kDa) were expressed and purified as described (Carrasco et al., 2005; Gándara et al., 2017; Torres et al., 2019a,b,c; Moreno-Del Alamo et al., 2020). Cells bearing a His-tagged RadA/Sms or DisA variant were recombination proficient and apparently as capable of repairing DNA damage as *wt* cells (Gándara et al., 2017). RadA/Sms or DisA and their mutant variants have been purified using the same protocol used for the *wt* protein (Torres et al., 2019a,c). Purified DisA shows traces of a slow-moving band of ∼42 kDa that corresponds to c-di-AMP-bound DisA (Gándara et al., 2017). The purified proteins and their mutant variants lack any protease, exonuclease or endonuclease activity in pGEM3 Zf(+) ssDNA or dsDNA in the presence of 5 mM ATP and 10 mM Mg(CH_3_COO)_2_. The corresponding molar extinction coefficients for RadA/Sms, DisA, PcrA, and RecA were calculated as 24,930; 22,350; 70,375; and 15,200 M^–1^ cm^–1^, respectively, at 280 nm, as described (Carrasco et al., 2005). Protein concentration was determined using the above molar extinction coefficients. The concentrations of DisA (and its mutant variants), RadA/Sms (and its mutant variants), PcrA, and RecA are expressed as moles of monomers. In this study, experiments were performed under optimal RecA conditions in buffer A (50 mM Tris-HCl pH 7.5, 1 mM DTT, 80 mM NaCl, 10 mM Mg(CH_3_COO)_2_, 50 μg/ml bovine serum albumin [BSA], and 5% glycerol).

The oligonucleotides used for the construction of the DNA substrates are listed in Supplementary Table 1. The 3′-tailed HJ DNA was assembled by annealing J3-1, J3-2-110, J3-3, and J3-4, the 3′-fork DNA by annealing 170, 171, and 173, and the 5′-fork DNA by annealing 170, 172, and 173. The substrates were gel purified as described (Ayora et al., 2004; McGregor et al., 2005) and stored at 4°C. In the cartoon representation of substrates, the complementary strands are denoted in solid lines, and the non-complementary regions in dotted lines. The labeled strand is represented in gray color. The 3′ end is depicted as the half of an arrowhead. DNA concentrations were established using the molar extinction coefficients of 8,780 and 6,500 M^–1^ cm^–1^ at 260 nm for ssDNA and dsDNA, respectively, and are expressed as moles of nucleotides (nt).

*In vitro* protein-protein interaction was assayed using His-tagged DisA, His-RadA/Sms and native RecA (1.5 μg). Combinations of proteins in buffer B (50 mM Tris-HCl pH 7.5, 50 mM NaCl, 10 mM MgCl_2_, 5% glycerol) containing 20 mM imidazole were loaded onto 50-μl Ni^2+^ microcolumns at room temperature. Then, the Ni^2+^ columns were sequentially washed with buffer B containing increasing concentrations of NaCl (from 100 to 200 mM). Finally, the retained proteins were eluted with 50-μl of Buffer B containing 1 M NaCl and 400 mM imidazole. The proteins were separated by 17.5% (RecA-RadA/Sms) or 10% (RecA-DisA) SDS-PAGE and gels were stained with Coomassie Blue.

### ATP Hydrolysis Assays

The ATP hydrolysis activity of RecA or RadA/Sms was assayed via an NAD/NADH coupled spectrophotometric enzymatic assay (Yadav et al., 2012). The rate of ATP hydrolysis was measured in buffer A containing 5 mM ATP and an ATP regeneration system (620 μM NADH, 100 U/ml lactic dehydrogenase, 500 U/ml pyruvate kinase, and 2.5 mM phosphoenol-pyruvate) for 30 min at 37°C (Yadav et al., 2012). The order of addition of circular 3,199-nt pGEM3 Zf(+) ssDNA (cssDNA, 10 μM in nt) and purified proteins is indicated in the text. Data obtained from A_340_ absorbance were converted to ADP concentrations and plotted as a function of time (Yadav et al., 2012). Accepting that RecA, *wt* RadA/Sms or its mutant variant RadA/Sms C13A operates mostly under steady-state conditions, the maximal number of substrate-to-product conversion per unit of time for a 1 μM of protein monomer (k_cat_) was measured. *t*-Tests were applied to analyze the statistical significance of the data.

In the presence of 10 mM Mg^2+^ concentrations (buffer A), the cssDNA adopts secondary structures with single (mimicking a non-replicated fork) and double-hairpin motifs (analogous to a HJ). The size site of RecA and RadA/Sms on ssDNA are 3 and ∼30-nt, respectively, and of DisA on branched structures of ∼45-bp (Cox, 2007; Chen et al., 2008; Yadav et al., 2014; Kowalczykowski, 2015; Torres et al., 2021).

### c-di-AMP Formation

c-di-AMP formation was analyzed using [α-^32^P]-ATP and thin-layer chromatography (TLC) as described (Witte et al., 2008; Gándara et al., 2017). Reactions were performed at 37°C using a range of protein concentrations in buffer C (50 mM Tris-HCl pH 7.5, 50 mM NaCl, 1 mM DTT, 10 mM MgCl_2_, 50 μg/ml BSA, 0.1% Triton, 5% glycerol) containing 100 μM ATP (at a ratio of 1:2,000 [α^32^P]-ATP:ATP). The order of addition of circular 3,199-nt pGEM3 Zf(+) ssDNA (10 μM in nt) and purified proteins is indicated in the text. After a 30 min incubation, the reactions were stopped by adding 50 mM EDTA. 2 μl of each reaction were spotted onto 20 × 20 cm TLC polyethyleneimine cellulose plates and run for about 2 h in a TLC chamber containing running buffer D [1:1 (v/v) 1.5 M KH_2_PO_4_ (pH 3.6) and 70% ammonium sulfate]. Dried TLC plates were analyzed by phosphor-imaging and spots were quantified using ImageJ (NIH). *t*-Tests were applied to analyze the statistical significance of the data.

### DNA Unwinding Assays

The different [γ-^32^P]-forked DNA substrates used were incubated with increasing concentrations of RadA/Sms or its mutant variants, RecA or DisA, for 15 min at 30°C in buffer A containing 2 mM ATP in a 20-μl volume as previously described (Ayora et al., 2002). The reactions were deproteinized by phenol-chloroform, DNA substrates and products were precipitated by NaCl and ethanol addition, and subsequently separated using 6% (w/v) polyacrylamide gel electrophoresis (PAGE). Gels were run and dried prior to phosphor-imaging analysis, as described above. The bands were quantified using ImageJ (NIH). *t*-Tests were applied to analyze the statistical significance of the data.

## Results

### DisA Competes With RadA/Sms C13A for ssDNA Binding

To understand the interplay of RadA/Sms and DisA, we measured the ATP hydrolysis of RadA/Sms or its RadA/Sms C13A mutant variant in the C4 motif in the absence or presence of the 3,199-nt long circular ssDNA (cssDNA). Under the experimental condition used (see section “Materials and Methods”) no DisA contribution to ADP production is detected (Witte et al., 2008; Torres et al., 2019a,c).

**FIGURE 2:**
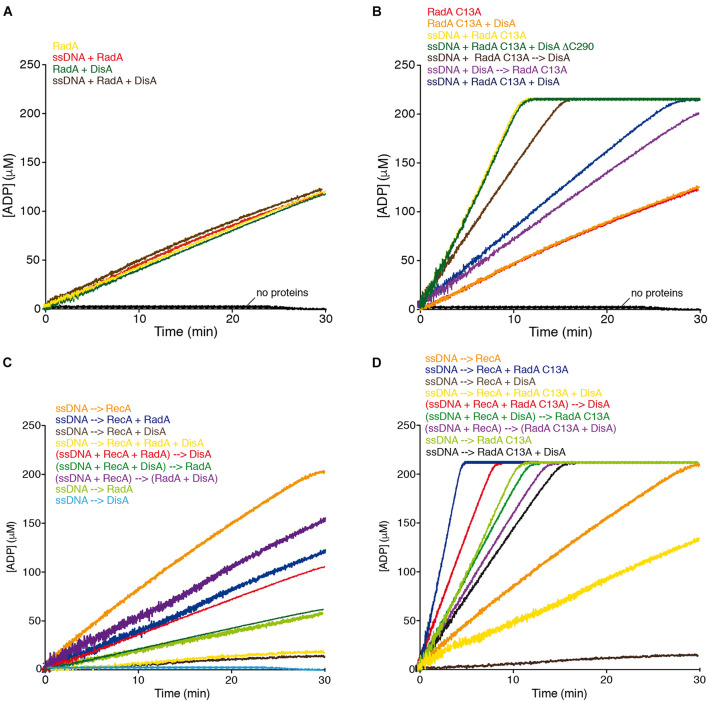
DisA inhibits RadA/Sms ATPase activity, and with RadA/Sms competitively reduce RecA-mediated ATP hydrolysis. **(A)** RadA/Sms-mediated ATP hydrolysis in the presence of DisA. Reactions had RadA/Sms (400 nM), DisA (500 nM), and when indicated cssDNA (10 μM in nt) in buffer A. **(B)** cssDNA was incubated with RadA/Sms C13A (400 nM) and DisA or DisA ΔC290 (500 nM), or cssDNA was pre-incubated with RadA/Sms C13A or DisA (5 min at 37°C), and then DisA or RadA/Sms C13A were added in buffer A. **(C)** cssDNA was incubated with RecA (800 nM), RadA/Sms (200 nM) or DisA (100 nM) or with RecA, RadA/Sms and DisA, or with RecA and RadA/Sms, or with RecA and DisA; or cssDNA was preincubated with RecA, or RecA and RadA/Sms or RecA and DisA (5 min at 37°C), then RadA/Sms, DisA or both were added in buffer A. **(D)** cssDNA was incubated with RecA (800 nM), RadA/Sms C13A (200 nM) or DisA (200 nM), or with RecA, RadA/Sms C13A and DisA, or with RecA and RadA/Sms C13A, or with RecA and DisA, or with RadA/Sms C13A and DisA; or cssDNA was pre-incubated with RecA, or with RecA and RadA/Sms C13A, or with RecA and DisA (5 min at 37°C), and then DisA, RadA/Sms C13A or both were added in buffer A. Buffer A contains the ATP regeneration system. Reactions were started by addition of ATP (5 mM), and the ATPase activity was measured (30 min at 37°C). All reactions were repeated three or more times with similar results. A representative graph is shown here, and quantifications of ATP hydrolyzed are shown in the main text as the mean ± SD of > 3 independent experiments.

RadA/Sms and RadA/Sms C13A hydrolyze ATP with similar efficiency in the absence of cssDNA (k_cat_ of 9.66 ± 0.2 and 9.60 ± 0.4 min^–1^, respectively). Addition of cssDNA significantly enhanced (∼5 fold) the rate of ATP hydrolysis of the latter (k_cat_ of 49.1 ± 0.4 min^–1^) (Torres et al., 2019b). Since RadA/Sms and RadA/Sms C13A physically interact with DisA at branched intermediates (Torres et al., 2019c), we tested if DisA has any effect on the ATPase activity of RadA/Sms. In the absence of DNA, the ATPase activity of *wt* RadA/Sms or RadA/Sms C13A (400 nM) was neither stimulated nor impaired by the addition of DisA (500 nM) (k_cat_ of 9.65 ± 0.2 and 9.63 ± 0.2 min^–1^, *p* > 0.1) (Figure 2A, green vs. yellow line and Figure 2B, orange vs. red line, Supplementary Figure 1A).

In the presence of cssDNA (10 μM in nt), addition of DisA significantly inhibited (∼2.5-fold, *p* < 0.01) the ATPase activity of RadA/Sms C13A (k_cat_ of 20.0 ± 0.5 min^–1^) (Figure 2B, yellow vs. blue line, Supplementary Figure 1B), suggesting that DisA either competes with RadA/Sms C13A for binding to cssDNA, or inhibits its ssDNA-stimulated ATPase activity by a protein-protein interaction. To evaluate that, the effect of the order of protein addition was analyzed. The rate of ATP hydrolysis was reduced (k_cat_ of 32.7 ± 0.2 min^–1^, *p* < 0.05) when a preformed RadA/Sms C13A-cssDNA complex was incubated with DisA (Figure 2B, brown vs. yellow line, Supplementary Figure 1B), but if RadA/Sms C13A was added to a preformed DisA-cssDNA complex, the ATPase activity was further inhibited (k_cat_ of 18.7 ± 0.5 min^–1^, *p* < 0.01) (Figure 2B, purple vs. yellow line, Supplementary Figure 1B). This suggests that DisA competes with RadA/Sms C13A for ssDNA binding. To confirm this, *wt* DisA was replaced by DisA ΔC290. This mutant variant, which lacks the DNA binding domain, still interacts with RadA/Sms C13A and its DAC activity is inhibited by RadA/Sms C13A (Torres et al., 2019a). DisA ΔC290 (500 nM) did not affect the ATP hydrolysis rate of RadA/Sms C13A (k_cat_ of 48.9 ± 0.5 min^–1^, *p* > 0.1) (Figure 2B, green vs. yellow line, Supplementary Figure 1B), confirming that DisA competes with RadA/Sms C13A for binding to cssDNA.

To test whether DisA inhibits non-specifically the activity of other ssDNA-dependent ATPases, the PcrA enzyme was chosen, because both PcrA and DisA inhibit the ATPase activity of RecA (Park et al., 2010; Torres et al., 2019a), and both PcrA and RadA/Sms act at stalled or reversed forks (Torres et al., 2019b; Moreno-Del Alamo et al., 2020). In the presence of saturating DisA concentrations relative to cssDNA (1 DisA/12-nt), the ATPase activity of PcrA (1 PcrA/660-nt) did not significantly vary (k_cat_ of 1750 ± 382 min^–1^ vs. 1722 ± 332 min^–1^, *p* > 0.1) (Supplementary Figure 2A, red vs. blue line). Furthermore, DisA neither affects PriA-dependent re-initiation of DNA replication nor DNA replication elongation using a reconstituted *in vitro* DNA replication system (Raguse et al., 2017). These observations confirm that the inhibition of the ATPase activity of RadA/Sms C13A is a genuine and specific DisA activity.

### RecA-RadA/Sms Complexes Show a Higher Stability Than RecA-DisA Complexes to Ionic Strength

DisA and RadA/Sms interact with and limit RecA activities (Torres et al., 2019a,c). To study the protein-protein interplay, we should know the relative concentration of the players and the strength of such protein-protein interactions. In unperturbed exponentially growing cells, RecA is abundant [∼4,000 RecA monomers/colony forming unit (CFU), ∼5.5 μM)], whereas DisA and RadA/Sms are less abundant proteins (∼600 DisA monomers/CFU, ∼800 nM, and ∼500 RadA/Sms monomers/CFU, ∼700 nM) (Cardenas et al., 2012; Muntel et al., 2014; Raguse et al., 2017). DisA and RadA/Sms, however, crystallize as octamers and hexamers, respectively, suggesting that their predicted amount is even smaller (Witte et al., 2008; Marie et al., 2017). Furthermore, upon DNA damage, the RecA level increases 5–6-fold as part of the SOS response, and the DisA pool increases ∼2.5-fold as part of the cell envelope stress response (Au et al., 2005; Eiamphungporn and Helmann, 2008; Cañas et al., 2011). Since cells bearing the His-tagged DisA or RadA/Sms proteins apparently are as capable of repairing DNA damage as *wt* cells (Torres et al., 2019b) and thus the tagged protein variants seem to function as the *wt* ones, we used His-tagged DisA or RadA/Sms bound to a Ni^2+^ matrix to retain native RecA and then evaluate the strength of such protein-protein interactions.

RecA (predicted mass of 38.0 kDa) migrates with an expected mass of ∼41.5 kDa (Supplementary Figure 3A, lane 1), and it is not trapped in the Ni^2+^ matrix (Torres et al., 2019c). His-tagged DisA, which has a predicted mass of 40.7 kDa contains traces of His-DisA bound to c-di-AMP (expected mass of 41 kDa) (Supplementary Figure 3A, lane 2; Gándara and Alonso, 2015).

RecA was pre-incubated with His-tagged DisA (5 min 37°C), and the mix was loaded onto a 50-μl Ni^2+^ matrix equilibrated in buffer B. Most RecA was retained in the DisA-bound Ni^2+^ matrix in the presence of 100 mM NaCl (Supplementary Figure 3A, lanes 3–4). RecA was eluted (E) in the presence of 150 mM NaCl, and traces of RecA facilitated the release of equimolar amounts of DisA from the matrix at 200 mM NaCl (Supplementary Figure 3A, lanes 5–6). Finally, when DisA bound to the matrix was competitively eluted with buffer B containing 400 mM imidazole and 1 M NaCl, no RecA was observed (Supplementary Figure 3A, lane 7).

Similarly, RecA was pre-incubated with His-tagged RadA/Sms (predicted mass 50.3 kDa) (5 min 37°C) and the mix was loaded onto a 50-μl Ni^2+^ matrix equilibrated with buffer B. RadA/Sms retained RecA in the Ni^2+^ matrix up to 200 mM NaCl. Both RadA/Sms and RecA eluted with buffer B containing 400 mM imidazole and 1 M NaCl (Supplementary Figure 3B, lanes 4–7). It is likely that a higher ionic strength is necessary to disrupt a RadA/Sms-RecA complex, when compared to the DisA-RecA complex.

### DisA and RadA/Sms Reduce the ATPase of RecA in a Mutually Exclusive Manner

RecA cooperatively binds ssDNA to form nucleoprotein filaments, with a site size of 1 RecA/3-nt (Chen et al., 2008). The ATPase activity of RecA, which is not required for homology search and strand exchange, might improve the efficiency of homology search, increase RecA filament continuity or the rate of release of RecA-ssDNA filaments from metastable search intermediates and regulate the processing of branched intermediates (Cox, 2007; Kowalczykowski, 2015). Thus, the kinetic of ssDNA-dependent ATP hydrolysis throughout the RecA filament is considered as an indirect readout of its nucleation and polymerization onto cssDNA (Cox, 2007; Kowalczykowski, 2015). DisA or RadA/Sms limits the ATPase activity of RecA, and as discussed previously, might enhance the stability of RecA⋅ATP assembled on ssDNA (Torres et al., 2019a,b).

To test whether DisA and RadA/Sms differentially regulate RecA nucleation and filament growth in concert or in a mutually exclusive manner, ATPase assays, which provide a real time view of the reaction progress, were used. RecA or RadA/Sms hydrolyzes ATP with a k_cat_ of 9.6 ± 0.4 and of 9.65 ± 0.2, respectively (Figure 2C, orange and light green lines, Supplementary Figure 1C; Torres et al., 2019b). RadA*_*Eco*_* also hydrolyzes ATP with a similar k_cat_ to that of RecA*_*Eco*_* (Cooper and Lovett, 2016).

The simultaneous addition of cssDNA, DisA (1 DisA/100-nt), RadA/Sms (1 RadA/Sms/50-nt), and RecA (1 RecA/12.5-nt) significantly blocked the maximum rate of ATP hydrolysis (k_cat_ of 1.0 ± 0.1 min^–1^, *p* < 0.01) (Figure 2C, yellow line, Supplementary Figure 1C), to levels comparable to the reaction mixture lacking RadA/Sms (Figure 2C, brown vs. yellow line). To understand the contribution of each protein, the order of protein addition was varied. Addition of DisA to preformed RecA-ssDNA-RadA/Sms complexes reduced the ATP hydrolysis rate (k_cat_ of 3.5 ± 0.3 min^–1^) to a level comparable to the reaction mixture lacking DisA (k_cat_ of 4.0 ± 0.3 min^–1^) (*p* > 0.1) (Figure 2C, red vs. dark blue line, Supplementary Figure 1C). On the other hand, addition of RadA/Sms to preformed RecA-ssDNA-DisA complexes inhibited the maximal rate of ATP hydrolysis (k_cat_ of 1.7 ± 0.2 min^–1^), although in a slightly less manifest way than when RadA/Sms was omitted (k_cat_ of 0.9 ± 0.1 min^–1^). Finally, when cssDNA was pre-incubated with RecA (5 min at 37°C), to allow nucleation, and then RadA/Sms and DisA were added, the maximum ATP hydrolysis rate was only moderately reduced (k_cat_ of 5.7 ± 0.3 min^–1^) (Figure 2C, purple line, Supplementary Figure 1C), suggesting that a RadA/Sms-DisA interplay renders a fraction of RecA free to hydrolyze ATP. Alternatively, the ssDNA-independent RadA/Sms ATPase activity might account for this small discrepancy (Figure 2C, dark brown line).

To further study this protein interplay, RadA/Sms was replaced by RadA/Sms C13A (k_cat_ of 49.1 ± 0.4 min^–1^), which fails to interact with RecA. Indeed, when incubated with RecA, neither the ATPase activity of RecA nor that of RadA/Sms C13A was inhibited (RecA + RadA/Sms C13A k_cat_ of 54.2 ± 0.4 min^–1^, Figure 2D, blue line; Torres et al., 2019b). When DisA, RadA/Sms C13A and RecA were simultaneously added to cssDNA, the rate of ATP hydrolysis was significantly reduced (k_cat_ of 7.4 ± 0.4 min^–1^, *p* < 0.01), but not blocked as it was observed in the absence of RadA/Sms C13A (Figure 2D, yellow vs. brown lines, Supplementary Figure 1D). This inhibition, however, was ameliorated when limiting DisA was added to preformed RecA-ssDNA-RadA/Sms C13A complexes (k_cat_ of 30 ± 0.7 min^–1^) (Figure 2D, red line vs. blue line). Moreover, the inhibition was partially reversed when RadA/Sms C13A was added to preformed DisA-ssDNA-RecA complexes (k_cat_ of 18.2 ± 0.3 min^–1^). Here, the activity was similar to that of RadA/Sms C13A alone (Figure 2D, dark vs. light green lines, Supplementary Figure 1D). When RadA/Sms C13A and DisA were pre-incubated before being added to preformed ssDNA-RecA complexes (k_cat_ of 16.1 ± 0.4 min^–1^), the activity resembled the sum of that of RadA/Sms C13A inhibited by DisA plus that of RecA alone (Figure 2D, purple vs. black and orange lines, Supplementary Figure 1D).

From the data presented here, it is likely that: (i) DisA blocks the ATPase activity of RecA, and addition of RadA/Sms does not reverse this blockage; (ii) a preformed RadA/Sms-ssDNA-RecA complex reduces the maximal rate of ATP hydrolysis of RecA, but addition of DisA shows no additive effect; (iii) the DisA and RadA/Sms activities on RecA-mediated ATP hydrolysis are mutually exclusive; and (iv) DisA interacts with and inhibits the ATPase activity of RadA/Sms C13A and RecA, and both become partially insensitive to DisA action in the presence of the other interacting partner (RecA or RadA/Sms). We consider unlikely that DisA-mediated inhibition is caused by c-di-AMP or ATP titration instead of by a protein-protein interaction, because the DisA D77N mutant variant, which does not synthesize c-di-AMP, still inhibits the ATPase activity of RecA (Torres et al., 2019c).

### DisA and RecA Inhibit RadA/Sms-Mediated Unwinding of a 3′-Fork DNA

**FIGURE 3:**
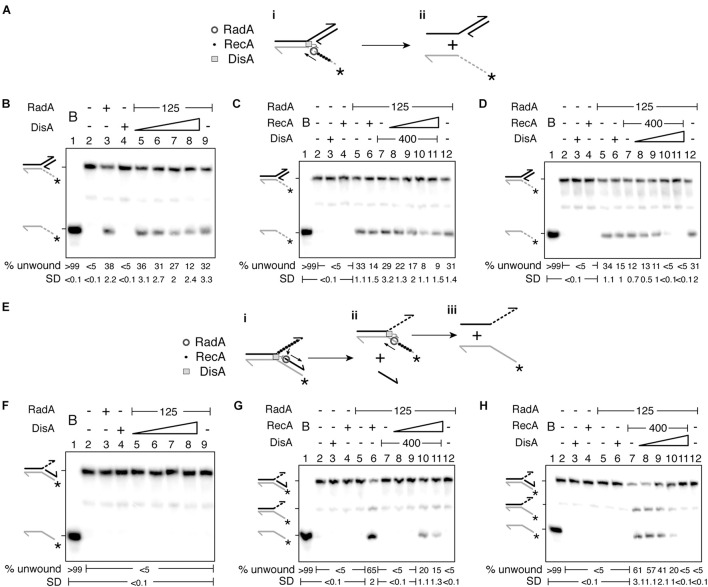
DisA inhibits RadA/Sms unwinding. **(A)** Cartoon illustrating how RadA/Sms unwinds a 3′-fork DNA substrate in the presence of DisA and RecA. RadA/Sms unwinds the substrate from its 5′ tail (i-ii), originating a 5′-tailed intermediate, but DisA bound at the junction and RecA bound at the ssDNA 5′tail inhibit RadA/Sms-mediated unwinding. **(B–D)** Helicase assays with 3′-fork DNA. The DNA was incubated with a fixed amount of RadA/Sms (and RecA in **D**) and increasing concentrations of DisA (100–800 nM) **(B,D)**; or with a fixed amount of RadA/Sms and DisA and increasing concentrations of RecA (50–400 nM) **(C)**. **(E)** Cartoon illustrating how RadA/Sms unwinds a 5′-fork DNA substrate in the presence of DisA and RecA. RecA filamented at the ssDNA 3′ tail loads RadA/Sms at the junction on the nascent lagging strand. Then, RadA/Sms unwinds the substrate (i,ii), originating a forked intermediate and the nascent lagging strand, but DisA bound at the junction inhibit RadA/Sms-mediated unwinding (i-ii). **(F–H)** Helicase assays with 5′-fork DNA. The DNA was incubated with a fixed amount of RadA/Sms (and RecA in **H**) and increasing concentrations of DisA **(F,H)**; or with a fixed amount of RadA/Sms and DisA and increasing concentrations of RecA **(G)**. Reactions were done in buffer A containing 2 mM ATP (15 min, 30°C), and after deproteinization the substrate and products were separated by 6% PAGE and visualized by phosphor imaging. The quantification values of unwound DNA and the SD of > 3 independent experiments are documented. B, boiled DNA substrate; − and +, absence and presence of the indicated protein; * and gray color, the labeled strand; half of an arrowhead, the 3′ end.

Previously it has been shown that RadA/Sms or RadA*_*Spn*_* unwinds a 3′-fork DNA (substrate with a fully synthesized leading-strand and no synthesis in the lagging-strand) by moving in the 5′→3′ direction (Marie et al., 2017; Torres et al., 2019b). Since DisA affects the ssDNA-stimulated ATPase activity of RadA/Sms C13A (Figures 1A,B), and RecA competes with RadA/Sms for binding to the 5′-tail of the 3′-fork DNA (Figure 3A; Torres et al., 2019b), we tested how DisA and RecA regulate RadA/Sms-mediated unwinding.

Increasing DisA concentrations (100–800 nM) significantly reduced (by 2–3-fold, *p* < 0.05) the unwinding activity of RadA/Sms or RadA/Sms C13A of a 3′-fork DNA substrate (Figure 2B and Supplementary Figure 4A, lanes 5–8). To test whether this inhibition is solely due to a competition for DNA binding, the DisA ΔC290 variant was used. DisA ΔC290 (100–800 nM) inhibited DNA unwinding to a similar extent to *wt* DisA does (*p* > 0.1) (Supplementary Figure 4B, lanes 5–8). This implies that the inhibition of RadA/Sms-mediated unwinding is not caused by a competition for DNA binding with DisA, as observed for the ssDNA-dependent ATPase activity of RadA/Sms C13A, but by a direct protein-protein interaction.

Then, it was tested whether RecA and DisA regulate RadA/Sms-mediated unwinding activity additively or in a mutually exclusive manner. When the 3′-fork DNA was incubated with increasing RecA concentrations (50–400 nM) and fixed amounts of DisA and RadA/Sms, the helicase activity of RadA/Sms was significantly reduced [by 4–5-fold (*p* < 0.01)] (Figure 3C, lanes 8–11). However, when the 3′-fork DNA was incubated with fixed RecA and RadA/Sms and increasing DisA concentrations, DNA unwinding was blocked at a higher DisA concentration (*p* < 0.01) (Figure 3D, lanes 8–11). When the expected stoichiometry of the reaction was analyzed, we assumed that a moderate excess of DisA limits the unzipping reaction, because a RadA/Sms [as hexamers]:DisA [as octamers]:RecA [as monomers] complex at a molar ratio of 1:2:20 reduces (by ∼4 fold), and at a molar ratio of 1:4:20 inhibits (by > 8 fold) RadA/Sms-mediated unwinding of the 3′-fork DNA substrate. A similar result was observed when DisA was substituted by DisA ΔC290 (Supplementary Figure 4C), confirming that DisA-mediated inhibition of RadA/Sms helicase activity is not simply due to competition for DNA binding. It seems that DisA and RecA affect RadA/Sms-mediated helicase activity in a nearly additive fashion. While RecA competes RadA/Sms for DNA binding, because RecA also inhibits RadA/Sms C13A-mediated unwinding (Figures 2A–D and Supplementary Figure 4A), DisA inhibits RadA/Sms helicase activity by a protein-protein interaction (Figures 2A,B and Supplementary Figures 4B,C). Alternatively, DisA may re-position RadA/Sms on the DNA, and the enzyme translocates away from the duplex junction.

### RecA Activates RadA/Sms-Mediated Unwinding of a 5′-Fork DNA, but DisA Inhibits It

We can envision that: (i) RadA/Sms bound to the 3′-tail of a 5′-fork DNA substrate (fully synthesized lagging-strand and no synthesis in the leading-strand) translocates away from the junction; and (ii) RecA bound to the 3′-tail of a 5′-fork DNA is necessary to activate RadA/Sms or RadA*_*Spn*_* to unwind its nascent lagging-strand (Figure 3E; Marie et al., 2017; Torres et al., 2019b). To test whether DisA re-positions RadA/Sms on the DNA, we have used this 5′-fork DNA. Increasing DisA or DisA ΔC290 concentrations did not activate RadA/Sms or RadA/Sms C13A to unwind the 5′-fork DNA substrate (Figure 3F and Supplementary Figures 4D,E, lanes 5–8). This suggests that DisA neither re-positions RadA/Sms to move toward the junction and unwind the substrate nor facilitates RadA/Sms-mediated unwinding upon binding to the 5′-fork DNA substrate.

Then, it was tested whether DisA regulates RecA activation of RadA/Sms on this non-cognate substrate or if DisA, by interacting with RecA and RadA/Sms, regulates the helicase activity of RadA/Sms (or RadA/Sms C13A). When the 5′-fork DNA substrate was incubated with a fixed DisA concentration, low RecA concentrations (50–100 nM) were not sufficient to activate RadA/Sms-mediated unwinding of a 5′-fork DNA substrate (Figure 3G, lanes 8–9). A higher RecA concentration (200 nM) was necessary to activate RadA/Sms-mediated unwinding (Figure 3G, lane 10). Under these conditions, RecA as a part of the RadA/Sms [hexamers]:DisA[octamers]:RecA [monomers] complex at molar ratios of 1:2:2.5 or 1:2:5 was not sufficient to activate RadA/Sms-mediated unwinding of the 5′-fork DNA substrate (Figure 3G, lanes 8–9). However, RecA at molar ratios of 1:2:10 or 1:2:20 activated RadA/Sms-mediated unwinding of the 5′-fork DNA substrate, albeit with 3- to 4-fold lower efficiency than when DisA was omitted (*p* < 0.01) (Figure 3G, lanes 10–11 vs. 6). It is likely that the unwinding activity of RadA/Sms is activated upon interacting with a discrete filament composed at least by 7–8 RecA monomers (Sussman et al., 2008; Yang et al., 2020).

When the 5′-fork DNA substrate was incubated with fixed RadA/Sms and RecA, and increasing concentrations of DisA, RecA as a part of the RadA/Sms[hexamers]:DisA[octamers]:RecA [monomers] complex at molar ratios of 1:0.6:20 or 1:1.2:20 activated RadA/Sms-mediated unwinding of the 5′-fork DNA substrate, but the RadA/Sms helicase activity was again inhibited at RadA/Sms [hexamers]:DisA[octamers]:RecA [monomers] molar ratios of 1:2.5:20 or 1:5:20 (Figure 3H, lanes 8–9 vs. 10–11). It is likely that DisA, upon interacting with RadA/Sms, counteracts the positive effect exerted by RecA filamented on the 3′-tail of the 5′-fork DNA substrate (Figure 3E) on the helicase activity of RadA/Sms (Figures 3G,H). Alternatively, DisA bound at the junction of the 5′-fork DNA competes RecA and abrogates the positive effect exerted by RecA on RadA/Sms helicase activity. To analyze these hypotheses, DisA was replaced by DisA ΔC290. When the 5′-fork DNA was incubated with fixed RadA/Sms and RecA and increasing DisA ΔC290 concentrations (100–800 nM), a molar excess of the latter still counteracted the positive effect produced by RecA over RadA/Sms helicase activity (Supplementary Figure 4F, lane 10). Accordingly, it is likely that DisA, by interacting with RadA/Sms or RecA, down regulates RecA-mediated activation of RadA/Sms-mediated unwinding of the nascent lagging-strand of a 5′-fork substrate (Figure 3H), a proper substrate for the loading of the replicative DNA helicase (Marians, 2018).

### RecA Activates RadA/Sms to Unwind a Reversed Fork With a Longer Nascent Leading-Strand, but DisA Inhibits It

**FIGURE 4:**
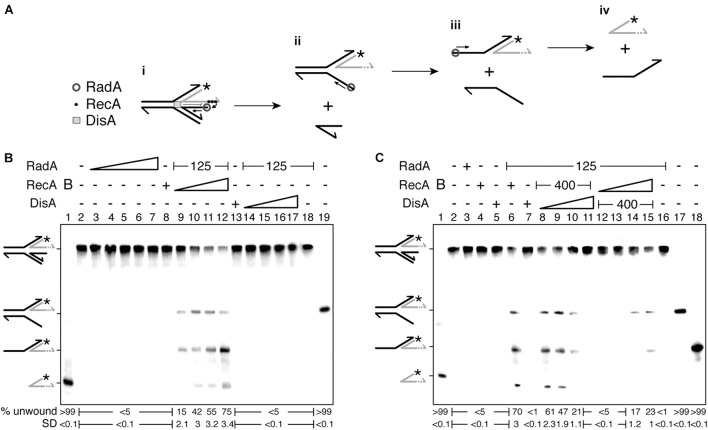
RecA facilitates RadA/Sms-mediated unwinding of a reversed fork with a longer nascent leading-strand, but DisA blocks it. **(A)** Cartoon illustrating how RecA promotes RadA/Sms-mediated unwinding of 3′-tail HJ DNA substrate. RecA filamented at the nascent leading-strand loads RadA/Sms at the nascent lagging-strand. Then, RadA/Sms unwinds the newly synthesized lagging-strand (i,ii), originating a 3′-fork intermediate, that is then further processed by RadA/Sms (ii–iv). **(B)** 3′-tail HJ DNA was incubated with increasing RadA/Sms (30–480 nM) concentrations, fixed RecA (400 nM) or DisA (800 nM) concentrations, or with a fixed concentration of RadA/Sms (125 nM) and increasing RecA (50–400 nM) or DisA (100–800 nM) concentrations; and the helicase activity measured. **(C)** 3′-tail HJ DNA was incubated with RadA/Sms (125 nM), RecA (400 nM), or DisA (800 nM), or with a fixed amount of RadA/Sms (125 nM) and RecA (400 nM) and increasing DisA concentrations (100–800 nM), or with a fixed RadA/Sms (125 nM) and DisA (400 nM) and increasing RecA concentrations (50–400 nM); and the helicase activity measured. Reactions were done in buffer A containing 2 mM ATP (15 min, 30°C), and after deproteinization the substrate and products were separated by 6% PAGE and visualized by phosphor imaging. The quantification values of unwound DNA and the SD of > 3 independent experiments are documented. B, boiled DNA substrate; − and +, absence and presence of the indicated protein; * and gray color, the labeled strand; half of an arrowhead, the 3′ end.

Previously it has been shown that RecA cannot activate RadA/Sms to unwind a remodeled fork with blunted-DNA ends (blunt-ended HJ structure) (Torres et al., 2019b). When a stalled replicating fork with a lagging-strand gap is remodeled, a HJ-like structure with a longer nascent leading-strand accumulates (Atkinson and McGlynn, 2009; Marians, 2018). This HJ-like structure with a 3′-tail was used to test whether RecA activates RadA/Sms to unwind the nascent lagging-strand (Figures 4Ai,ii), yielding a 3-way junction that upon spontaneous annealing might lead to a 3′-fork DNA, a restored fork preferentially bound by PriA.

To investigate the hypothesis, and whether the processing of a HJ-like structure with a longer 3′-nascent leading-strand is regulated by RecA and DisA, an artificial substrate (a HJ-like structure with the nascent leading-strand 30-nt longer than the nascent lagging-strand [3′-tail HJ]) was constructed (Figure 4Ai). The integrity of the 3′-tail HJ DNA substrate was confirmed by branch migrating it with the RecG enzyme as described (Torres et al., 2021). This short DNA substrate contains heterologous arms to prevent spontaneous branch migration, but the 5′-end of the template lagging-strand and then that of the template leading-strand are exposed to be unwound by RadA/Sms (see Figures 4Aii,iii). In contrast, *in vivo*, the parental strands of such reversed fork should not have available ends.

In the presence of the 3′-tail HJ DNA and increasing RadA/Sms concentrations (30–480 nM), fork regression (that would drive to the accumulation of two flayed structures) was not observed (Figure 4B, lanes 3–7). Similarly, RecA (400 nM) or DisA (800 nM) did not process this substrate (Figure 4B, lanes 8 and 13). In the presence of a fixed RadA/Sms and a limiting RecA (50 nM) concentration, RadA/Sms unwound the nascent lagging-strand, yielding a 3-way junction and a forked intermediate (Figures 4Ai–iii,B, lane 9). Fork regression was not observed, suggesting that RadA/Sms cannot regress a 3′-tail HJ DNA as described for RecG (Torres et al., 2021). We can envision that RecA nucleated on the nascent leading-strand of the 3′-tail HJ DNA interacts with and loads RadA/Sms at the nascent lagging-strand; then, RadA/Sms unwound it. This is followed by RadA/Sms binding to the 5′-tail of the 3-way junction, that is unwound yielding a forked intermediate (Figures 4Ai–iii,B, lane 9). At higher RecA concentrations (100–400 nM), RadA/Sms also unwound the forked intermediate to free the labeled nascent leading-strand (Figures 4Aiii,iv,B, lanes 10–12). When RecA was replaced by increasing DisA concentrations (100–800 nM), no unwinding was detected (Figure 4B, lanes 14–17).

To analyze whether DisA affects RecA-mediated activation of RadA/Sms to catalyze the unwinding of the nascent lagging-strand, DisA was added to the reaction (Figure 4C). In the presence of fixed RadA/Sms and RecA concentrations, increasing concentrations of DisA monomers (100–800 nM) significantly inhibited RadA/Sms-mediated unwinding of the 3′-tail HJ DNA (*p* < 0.01). Indeed, at a higher DisA molar excess (RadA/Sms [hexamers]:DisA[octamers]:RecA [monomers] at a molar ratio of 1:5:20) the RadA/Sms-mediated unwinding activity was blocked. However, with DisA at a molar ratio of 1:0.6:20, RadA/Sms efficiently unwound the 3′-tail HJ DNA substrate (Figure 4C, lane 8 vs. 11).

To analyze whether this blockage is reversible, and if it is due to a competition for ssDNA binding of DisA with RecA, fixed concentrations of RadA/Sms and DisA, and increasing concentrations of RecA (50–400 nM) were used. DisA inhibited RadA/Sms-mediated DNA unwinding of the 3′-tail HJ DNA substrate in the presence of low RecA concentrations (Figure 4C, lanes 12–13). The presence of RecA at a RadA/Sms [hexamers]:DisA[octamers]:RecA [monomers] molar ratio of 1:2.5:10 partially counteracted DisA, and RadA/Sms unwound the substrate, yielding the first intermediate. When RecA was added at a RadA/Sms [hexamers]:DisA[octamers]:RecA [monomers] molar ratio of 1:2.5:20, the second DNA intermediate was also observed (Figures 4Aiii,C, lanes 14–15). It is likely that: (i) RecA activates RadA/Sms to unwind the nascent lagging-strand, leading to fork restoration upon spontaneous pairing of the nascent leading-strand, and DisA downregulates the process; and (ii) RecA interacts with and competes DisA.

To test whether the inhibition on the helicase activity of RadA/Sms is a specific activity of DisA, RadA/Sms was replaced by PcrA. In the presence of increasing DisA concentrations, the helicase activity of PcrA did not significantly vary at a PcrA [monomers]:DisA[octamers] molar ratio of 1:0.8–1:6.6 (*p* > 0.1) (Supplementary Figure 2B). This confirms that the inhibition caused by DisA over RadA/Sms-mediated fork restoration is a genuine activity of DisA that works at about stoichiometric ratios.

### RecA Antagonizes RadA/Sms-Mediated Inhibition of DisA DAC Activity

DisA synthesizes c-di-AMP, but this synthesis is inhibited when DisA binds branched DNA intermediates, and in less extent ssDNA (Witte et al., 2008), or when DisA interacts with RadA/Sms (Zhang and He, 2013; Gándara et al., 2017). DisA-mediated c-di-AMP synthesis is blocked in the presence of both HJ DNA and RadA/Sms (Gándara and Alonso, 2015; Torres et al., 2019c). In rich medium, low c-di-AMP levels indirectly block cell proliferation until DNA damage is repaired or circumvented (see “Introduction”). In previous sections, we have gained insights in the global DisA-RadA/Sms-RecA interplay analyzing RecA and RadA/Sms activities. To further understand the protein-protein interactions, the DAC activity of DisA was measured in the presence of RadA/Sms and RecA.

In the presence of 10 mM Mg^2+^ and 100 μM ATP, DisA converts two ATP molecules into c-di-AMP (Witte et al., 2008; Gándara et al., 2017). Under the conditions used to detect [α-^32^P]-c-di-AMP, [α-^32^P]-ADP is poorly separated from the [α-^32^P]-ATP substrate (Torres et al., 2019b). RadA/Sms or its mutant variants (the Walker A RadA/Sms K104A or RadA/Sms C13A), at or above stoichiometric concentrations, interacted with and significantly inhibited DisA-mediated c-di-AMP synthesis (by ∼20-fold [*p* < 0.01]), but an excess of RecA did not affect DisA-mediated c-di-AMP synthesis (*p* > 0.1) (Figures 4A–C, lane 2 vs. 3 or 4).

To test whether RecA affects RadA/Sms-mediated inhibition of DisA DAC activity, fixed amounts of DisA and RadA/Sms (or its mutant variants) and increasing RecA concentrations were simultaneously added. Here, RadA/Sms or RadA/Sms K104A only reduced 3–4-fold (*p* < 0.01) the DAC activity of DisA. However, RadA/Sms C13A blocked the DAC activity of DisA even in the presence of RecA (Figures 4A–C, lane 11), suggesting that a RecA-RadA/Sms interaction is necessary to observe c-di-AMP synthesis restoration. Thus, it is likely that RecA counters RadA/Sms-mediated inhibition of DisA-mediated c-di-AMP synthesis.

**FIGURE 5:**
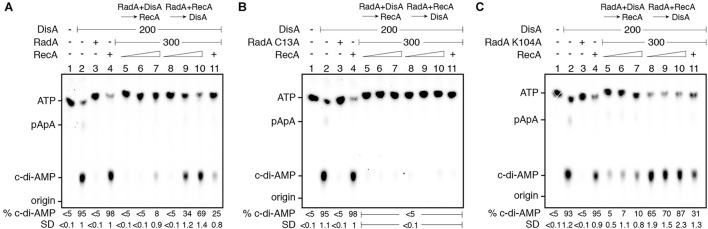
RadA/Sms inhibits DisA DAC activity, but RecA counters this negative effect. DisA (200 nM), or DisA (200 nM) and RadA/Sms **(A)**, RadA/Sms C13A **(B),** or RadA/Sms K104R **(C)** (300 nM), or DisA (200 nM) and RecA (1,600 nM), or DisA (200 nM), RecA (1,600 nM) and RadA/Sms **(A)**, RadA/Sms C13A **(B)**, or RadA/Sms K104R **(C)** (300 nM) were incubated in buffer C containing 100 μM [α^32^P]-ATP:ATP (30 min, 37°C). DisA (200 nM) was incubated with a fixed concentration of RadA/Sms **(A)**, RadA/Sms C13A **(B)**, or RadA/Sms K104R **(C)** (300 nM) (5 min, 37°C), and then increasing RecA concentrations (400–1,600 nM) were added in buffer C containing 100 μM [α^32^P]-ATP:ATP (30 min, 37°C). Fixed RadA/Sms **(A)**, RadA/Sms C13A **(B)**, or RadA/Sms K104R **(C)** (300 nM) and increasing RecA (400–1,600 nM) concentrations were pre-incubated (5 min, 37°C), and then a fixed amount of DisA (200 nM) was added in buffer C containing 100 μM [α^32^P]-ATP:ATP (30 min, 37°C). The substrate, intermediates and products were separated by TLC and quantified. The quantification values of c-di-AMP synthesis and the SD of > 3 independent experiments are documented. The position of [α^32^P]-ATP:ATP, linear pppA-pA (denoted as pApA), c-di-AMP and the origin are indicated.

To gain insight of the protein-protein interactions, the order of protein addition was varied. When DisA was pre-incubated with RadA/Sms or RadA/Sms K104A (5 min 37°C), and then increasing concentrations of RecA and ATP were added, the DAC activity of DisA was only moderately restored at a high RecA concentration (Figures 4A,C, lanes 5–7). As expected, RecA, which fails to interact with RadA/Sms C13A, did not antagonize the negative effect of RadA/Sms C13A on the preformed RadA/Sms C13A-DisA complexes (Figure 5B, lanes 5–7). When *wt* RadA/Sms or RadA/Sms K104A was pre-incubated with increasing RecA concentrations (5 min 37°C), and then DisA and ATP were added, the preformed RadA/Sms-RecA or RadA/Sms K104A-RecA complex marginally inhibited DisA-mediated c-di-AMP synthesis (*p* < 0.01) (Figures 4A,C, lanes 8–10). This confirms that RecA antagonizes the negative effect of RadA/Sms on DisA-mediated c-di-AMP synthesis. To confirm if the interaction of RecA with RadA/Sms is necessary, RadA/Sms was replaced by the RadA/Sms C13A mutant. When RecA was pre-incubated with RadA/Sms C13A (5 min at 37°C), and then ATP and DisA and were added, an excess of RecA did not counteract the inhibition of RadA/Sms C13A on DisA-mediated c-di-AMP synthesis (*p* > 0.1) (Figure 5B, lanes 8–10).

RecA efficiently nucleates on ssDNA (Cox, 2007; Kowalczykowski, 2015). Thus, to test whether a preformed RecA nucleoprotein filament further controls the inhibition of c-di-AMP production, the DAC activity of DisA was measured in the presence of ssDNA, RecA and RadA/Sms. The presence of RadA/Sms, RadA/Sms C13A or ssDNA (10 μM in nt) strongly inhibited DisA-mediated c-di-AMP synthesis (*p* < 0.01) (Supplementary Figures 5A,B). As described in the absence of ssDNA (Figures 5A,B), RecA cannot antagonize the negative effect of RadA/Sms and in major extent of RadA/Sms C13A when the RadA/Sms-ssDNA-DisA or RadA/Sms C13A-ssDNA-DisA complex was pre-assembled, but it can do it if DisA was added to the pre-assembled RadA/Sms-ssDNA-RecA complex (Supplementary Figures 5A,B). Here, the RecA-RadA/Sms interaction is confirmed to be relevant for DisA-mediated c-di-AMP synthesis recovery since RecA did not counteract the negative effect of RadA/Sms C13A when the RadA/Sms C13A-ssDNA-RecA complex was pre-assembled (Supplementary Figures 5A,B).

These data altogether suggest that: (i) RecA poorly counteracts the negative effect of RadA/Sms on c-di-AMP synthesis from preformed RadA/Sms-DisA complexes (Figure 5A, lanes 5–7); (ii) RecA efficiently counteracts the negative effect of RadA/Sms on DisA-mediated c-di-AMP synthesis when complexed with RadA/Sms (Figure 5A, lanes 8–10); (iii) the RecA-RadA/Sms interaction is necessary to antagonize the negative effect of RadA/Sms on DisA-mediated c-di-AMP synthesis (Figure 5B, lanes 5–11); and (iv) RecA is as efficient as RecA-ssDNA complexes to counter the negative effect of RadA/Sms as a part of a preassembled RadA/Sms-ssDNA-RecA complex.

## Discussion

Our results support a comprehensive role of DisA at the intersection between recombination and replication restart, by regulating RecA and RadA/Sms activities at branched intermediates (stalled or reversed forks) to prevent fork remodeling that should be pathological during spore revival (see “Introduction”). Collectively, the study presented here emphasizes the importance of timely and flexible responses of DisA to the formation and in the stability of remodeled stalled replication forks. Similarly, in eukaryotes, a temporal window is open to allow the access of homologous recombination functions at the stalled fork, and this process is also tightly controlled (Branzei and Foiani, 2010; Neelsen and Lopes, 2015; Quinet et al., 2017; Berti et al., 2020).

When the single genome of an inert mature haploid *B. subtilis* spore is damaged, unperturbed spore revival requires RecA, RecG, RadA/Sms and DisA, but not functions involved in end resection (as the RecJ ssDNA exonuclease in concert with a RecQ-like (RecS or RecQ) DNA helicase or the AddAB helicase/nucleases complex) (Vlasic et al., 2014; Raguse et al., 2017). Upon DNA damage, DisA pausing requires RecA during sporulation and DisA or RadA/Sms pausing in exponentially growing cells requires the accumulation of branched intermediates (as in Δ*recG* cells) (Gándara et al., 2017; Raguse et al., 2017). DisA and RadA/Sms may co-localize at those recombination intermediates (see section “Introduction”). Previously, it has been shown that during a replication stress DisA limits the activities of RecA and RecG, perhaps to gain time for damage removal (Torres et al., 2019a, 2021), and RecA activates RadA/Sms-mediated unwinding of non-cognate DNA substrates (Torres et al., 2019b). Here, we present for the first-time biochemical evidences of the interplay of DisA with RecA and RadA/Sms in the stability of nascent strands after replication fork stalling, and how these protein interactions support a spatio-temporal regulation of fork processing and may determine the selection of an appropriate DDT sub-pathway (Branzei and Foiani, 2010; Raguse et al., 2017; Marians, 2018).

When the replisome stalls, RecA bound to the lesion containing-gap interacts with and loads DisA and RadA/Sms at a stalled or reversed fork (Torres et al., 2019a,b). Then, RadA/Sms interacts with and blocks DisA-mediated c-di-AMP synthesis. Low c-di-AMP levels increase the production of (p)ppGpp, which in turn inhibits DNA primase and indirectly cell proliferation (Wang et al., 2007; Kriel et al., 2012).

**FIGURE 6:**
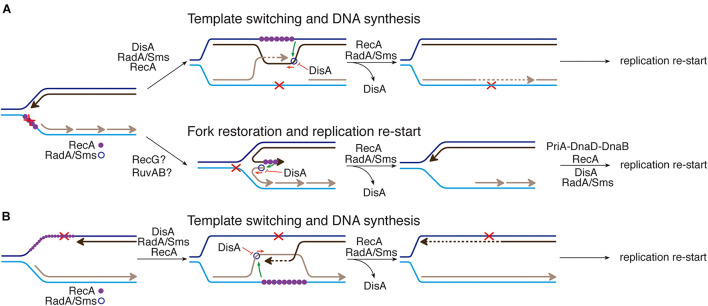
RecA, DisA and RadA/Sms interplay. **(A)** An unrepaired DNA lesion on the lagging-strand template (red cross) causes blockage of replication fork movement. RecA-bound to the lesion-containing gap suppresses DisA dynamic movement and loads RadA/Sms onto the forked or D-loop structure. (Template switching and DNA synthesis) RecA promotes D-loop formation and RadA/Sms mediates D-loop extension and the invaded strand primes DNA synthesis. DisA bound to D-loop DNA decreases c-di-AMP synthesis, that in turn increases (p)ppGpp synthesis, that inhibits cell proliferation. DisA suppresses RadA/Sms mediated D-loop extension and RecA reloads RadA/Sms in the complementary strand to promote D-loop disassembly. (Fork restoration and replication restart) RadA/Sms unwinds the nascent lagging-strand and allows the fork to be restored. DisA bound to forked DNA decreases c-di-AMP synthesis, that in turn increases (p)ppGpp synthesis, that inhibits cell proliferation. DisA suppresses RadA/Sms mediated DNA unwinding, and DnaD-PriA promote replication re-start. **(B)** An unrepaired DNA lesion on the leading-strand template (red cross) causes blockage of replication fork movement. RecA-mediated D-loop formation is regulated as depicted in **(A)**. DisA bound to D-loop or HJ DNA decreases c-di-AMP synthesis, that in turn increases (p)ppGpp synthesis, that inhibits cell proliferation.

Do the activities described here play a role to maintain cell survival? We propose that the interactions among RecA, DisA and RadA/Sms, which seem to be mutually exclusive (Figures 1–4), are crucial to circumvent a replication arrest and to postpone replication restart until the damage is removed. Dissection of the reaction uncovered several of the molecular details for the generation of a substrate for replication restart. RadA/Sms alone or in concert with RecA facilitates the processing of stalled or reversed forks by unwinding their nascent lagging-strand, but DisA limits RecA loading of RadA/Sms on the nascent lagging-strand and inhibits their ATPase activities (Figures 1, 3). It has been earlier proposed that if the damage is in the template lagging-strand, RecA invades and pairs the complementary nascent strands, resulting in a D-loop intermediate, in which the nascent leading-strand is used as a template for DNA synthesis of the nascent lagging-strand, contributing to circumvent the DNA damage (template switching) (Figure 6A, template switching) (Marians, 2018). Then, the offending lesion is removed by specialized pathways and RecA displaces RadA/Sms from the DisA-branched intermediate-RadA/Sms complexes by a protein-protein interaction to recover DisA-mediated c-di-AMP synthesis and reverse the cell proliferation inhibition, with subsequent replication restart (Figure 6A). Also during DSB repair, RecA bound to the displaced strand might load RadA/Sms at the D-loop to displace the invading strand as proposed for *Deinococcus radiodurans* RadA in concert with RecA (Slade et al., 2009). On the other hand, if any of the putative fork remodelers pushes backward the stalled fork with a lesion in the lagging-strand, a HJ with nascent leading-strand longer than the nascent lagging-strand may accumulate (Figure 6A, fork restoration; Atkinson and McGlynn, 2009; Neelsen and Lopes, 2015; Marians, 2018). Here, RecA filamented on the 3′-nascent leading-strand may activate RadA/Sms to restore a replication fork with a lagging-strand gap, but DisA limits fork restoration. Then, RecA displaces RadA/Sms from the DisA-HJ-RadA/Sms complexes by a protein-protein interaction to recover DisA-mediated c-di-AMP synthesis and reverse the cell proliferation inhibition.

When there is a lesion on the leading-strand template, the stalled fork may be converted into a HJ-like structure with a 5′-tail at the regressed nascent lagging-strand (Atkinson and McGlynn, 2009; Neelsen and Lopes, 2015; Marians, 2018). Here, both DisA and RecA may compete with RadA/Sms for binding to the template lagging-strand or the 5′-tail of a HJ DNA substrate, as earlier described (Torres et al., 2019b, 2021). Alternatively, template switching may occur, with the nascent lagging-strand serving as a template for the nascent leading-strand synthesis (Figure 6B, template switching; Branzei and Foiani, 2010). Then, the lesion on duplex DNA is removed/repaired, and DisA synthesizes c-di-AMP to indirectly free the DnaG primase (see Wang et al., 2007). Finally, PriA might recognize the 3′-end of the nascent leading strand of a 3′-fork DNA, and RecA and SsbA bound to ssDNA recruit DnaD and PriA, respectively (Costes et al., 2010; Sanders et al., 2010; Million-Weaver et al., 2015). Subsequently, pre-primosome (PriA-DnaD-DnaB) assembly may facilitate replication re-start (Sanders et al., 2010).

In summary, we propose that DisA limits the activities of RecA and RadA/Sms (Figure 6), and this is expected to occur when damaged template bases stall the replisome during spore revival. DisA, however, does not act as a protein block to the recruitment of replication proteins, because *in vitro* DisA does not affect PriA-dependent re-initiation of DNA replication, DNA replication elongation (Raguse et al., 2017), or the activities of the PcrA enzyme (Figures 2, 5 and Supplementary Figures 1A,B). The protein interplay described here might apply to other bacteria that encode these three proteins, like *M. tuberculosis*, which infects one-third of the world population and cause tuberculosis. Understanding the role of RecA, DisA, and RadA/Sms at stalled replication forks may provide strong mechanistic basis for potential DisA or RadA/Sms inhibitors to be used in *Mycobacterium* therapy.

## Data Availability Statement

The original contributions presented in the study are included in the article/Supplementary Material, further inquiries can be directed to the corresponding author/s.

## Author Contributions

RT and JA: designed the research, analyzed the data, writing-review, and editing. RT: performed the research. JA: writing-original draft and funding acquisition. Both authors contributed to the article and approved the submitted version.

## Conflict of Interest

The authors declare that the research was conducted in the absence of any commercial or financial relationships that could be construed as a potential conflict of interest.

## Publisher’s Note

All claims expressed in this article are solely those of the authors and do not necessarily represent those of their affiliated organizations, or those of the publisher, the editors and the reviewers. Any product that may be evaluated in this article, or claim that may be made by its manufacturer, is not guaranteed or endorsed by the publisher.
