# Supplementary material for: Bacillus subtilis RecA, DisA, and RadA/Sms Interplay Prevents Replication Stress by Regulating Fork Remodeling
Source: Front Microbiol. 2021 Nov 22;12:766897. doi: 10.3389/fmicb.2021.766897 (PMC8645862; doi:10.3389/fmicb.2021.766897)
Supplement: Supplementary file 1 [file Data_Sheet_1.docx]

Supplementary Material

*Bacillus subtilis* RecA, DisA and RadA/Sms interplay prevents replication stress by regulating fork remodeling

Rubén Torres and Juan C. Alonso^*^

Department of Microbial Biotechnology, Centro Nacional de Biotecnología, CNB-CSIC, 28049 Madrid, Spain

# Supplementary Table S1

| Oligonucleotides |
| --- |
| J3-1 (80-nt), CGCAAGCGACAGGAACCTCGAGAAGCTTCCGGTAGCAGCCTGAGCGG TGGTTGAATTCCTCGAGGTTCCTGTCGCTTGCG |
| J3-2-110 (110-nt), CGCAAGCGACAGGAACCTCGAGGAATTCAACCACCGCTCAACTC AACTGCAGTCTAGACTCGAGGTTCCTGTCGCTTGCGAAGTCTTTCCGGCATCGAT CGTAGCTATTT |
| J3-3 (80-nt), CGCAAGCGACAGGAACCTCGAGTCTAGACTGCAGTTGAGTCCTTGCTA GGACGGATCCCTCGAGGTTCCTGTCGCTTGCG |
| J3-4 (80-nt), CGCAAGCGACAGGAACCTCGAGGGATCCGTCCTAGCAAGGGGCTGCTA CCGGAAGCTTCTCGAGGTTCCTGTCGCTTGCG |
| 170 (60-nt), AGACGCTGCCGAATTCTGGCTTGGATCTGATGCTGTCTAGAGGCCTCCA CTATGAAATCG |
| 171 (30-nt), CGATTTCATAGTGGAGGCCTCTAGACAGCA |
| 173 (60-nt), AGCTCATAGATCGATAGTCTCTAGACAGCATCAGATCCAAGCCAGAATT CGGCAGCGTCT |
| 172 (30-nt), TGCTGTCTAGAGACTATCGATCTATGAGCT |

**Supplementary Table S1.** The nucleotide sequence of the oligonucleotides used is indicated in the 5´→3´polarity. Oligonucleotides were provided by Merck and their length indicated.

## Supplementary Figures


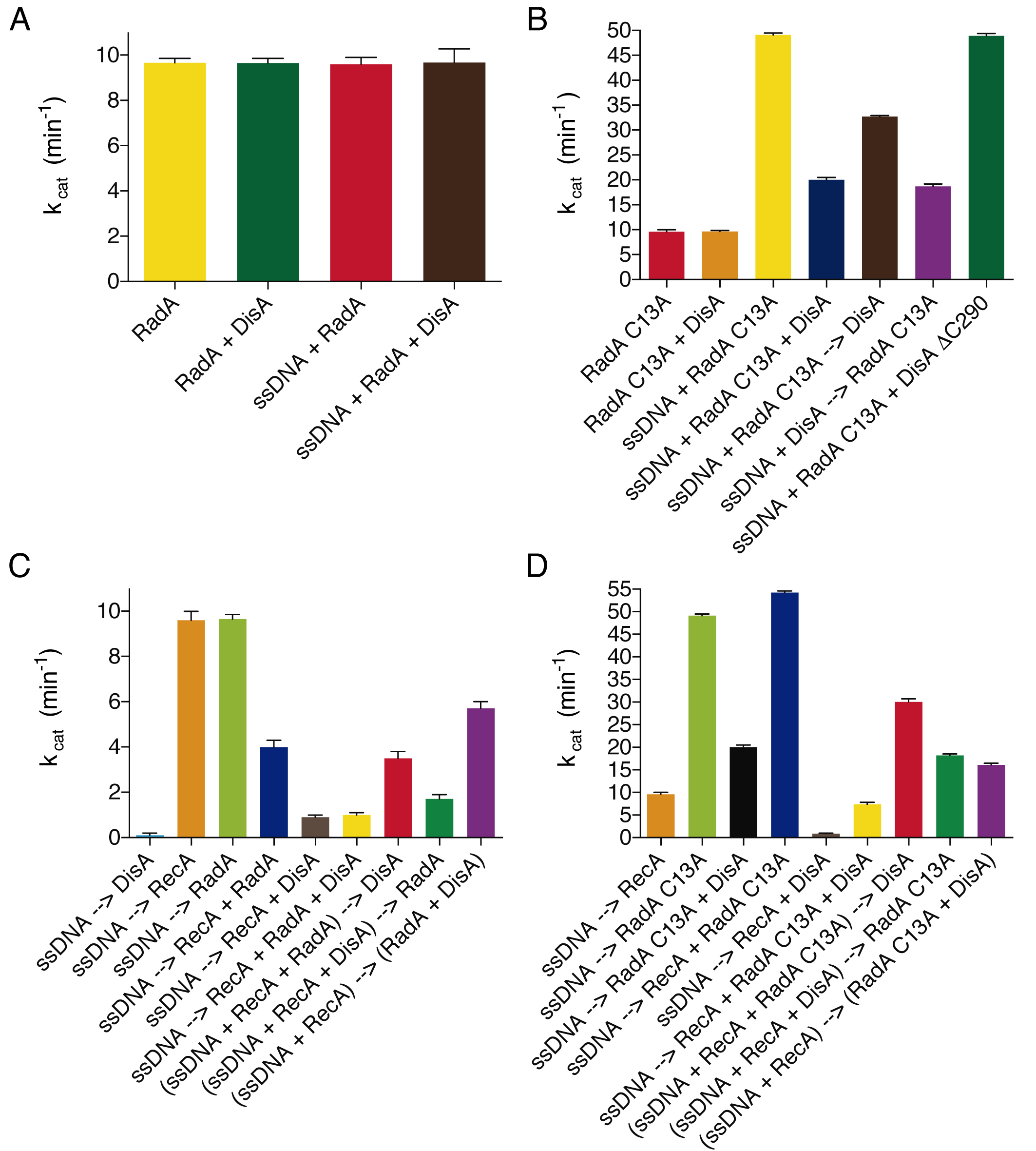


**Supplementary Figure S1.** The mean k_cat_ ± SD of the ATPase activities calculated from at least four independent experiments like those depicted in Figure 2A, 2B, 2C and 2D is represented in the bar graphs in panels A, B, C and D, respectively.


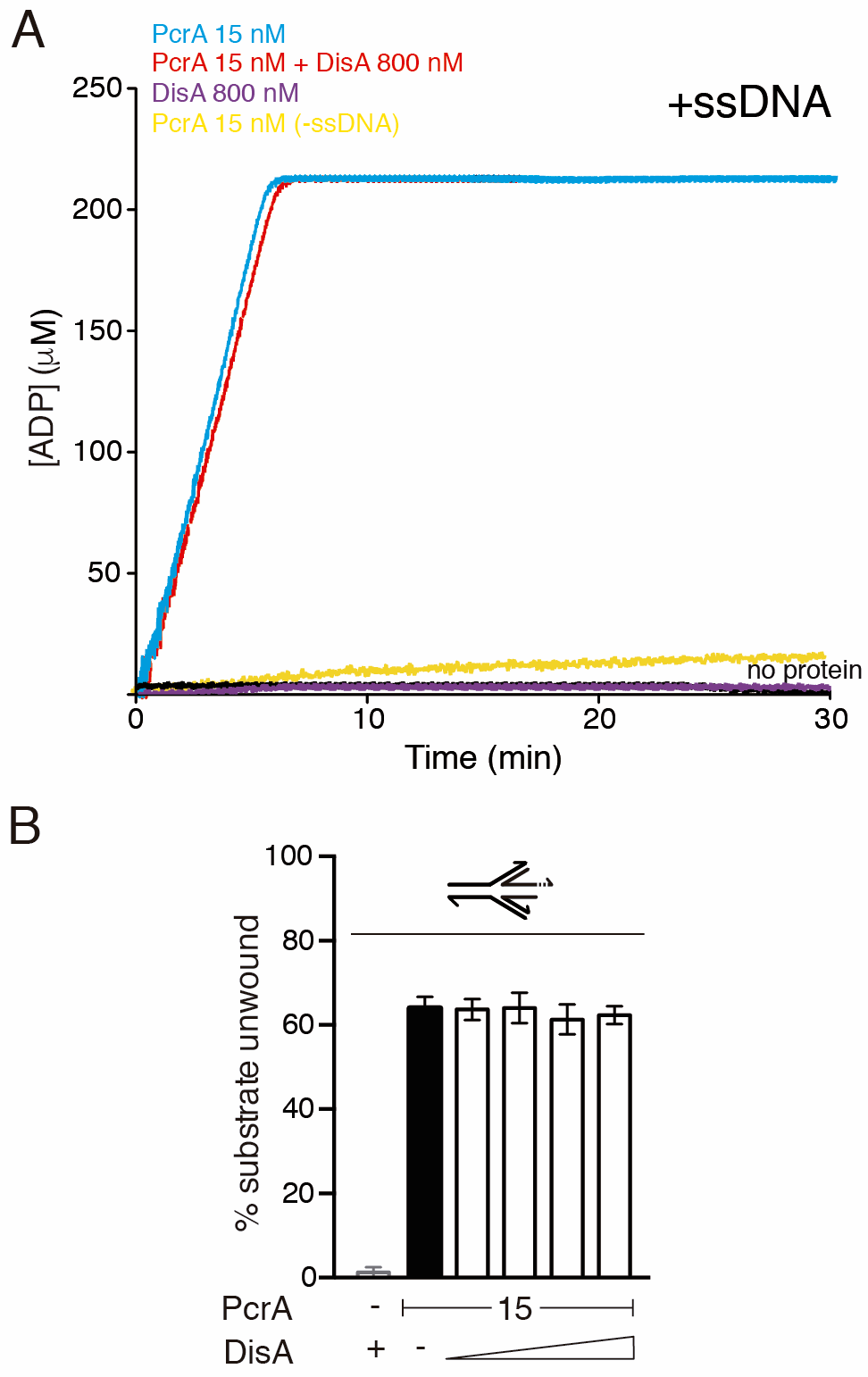


**Supplementary Figure S2.** The activities of PcrA are not regulated by DisA. (A) PcrA-mediated ATP hydrolysis in the presence of DisA. Reactions had PcrA (15 nM), DisA (800 nM) and circular 3,199-nt ssDNA (10 μM in nt) in buffer A containing ATP (5 mM) and the ATP regeneration system. Reactions were started by addition of ATP, and the ATPase activity was measured (30 min at 37 °C). All reactions were repeated three or more times with similar results. A representative graph is shown here, and quantifications of the ATP hydrolysis rates are shown in the main text as the mean ± SD of >3 independent experiments. (B) PcrA-mediated helicase assays with 3´-tail HJ DNA (a HJ-like structure with the nascent leading-strand 30-nt longer than the nascent lagging-strand). The DNA was incubated with PcrA (15 nM) and increasing concentrations of DisA (100-800 nM). Reactions were done in buffer A containing 2 mM ATP (15 min, 30ºC), and after deproteinization the substrate and products were separated by 6% PAGE and visualized by phosphor imaging. The quantification values of unwound DNA and the SD of >3 independent experiments are documented. Abbreviations: - and +, absence and presence of the indicated protein; half of an arrowhead, the 3’ end.


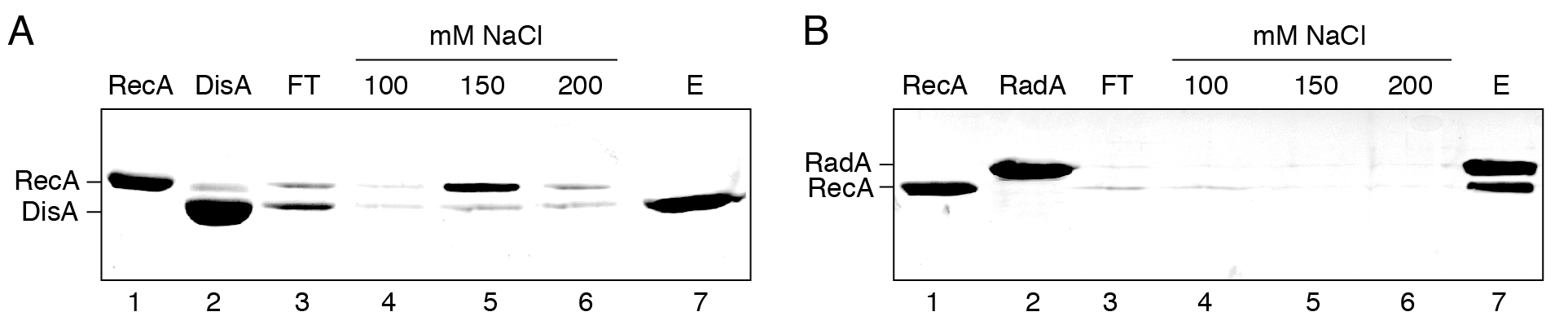


**Supplementary Figure S3.** RecA forms a stable complex with RadA/Sms, but not with DisA. His-tagged DisA (A) or His-tagged RadA/Sms (B) was incubated with RecA in buffer B (50 mM NaCl) (5 min, 37 ºC). Then, the mix was loaded onto a 50 μl Ni^2+^ matrix and the flow-through (FT) was collected. The Ni^2+^ matrix was washed once with buffer B and then with 500 μl of buffer B containing increasing NaCl concentrations. Finally, bound His-tagged DisA (A) or His-tagged RadA/Sms (B) was eluted (E) with Buffer B containing 1 M NaCl and 0.4 M imidazole. Experiments were repeated three or more times with similar results, and a representative gel is shown here.


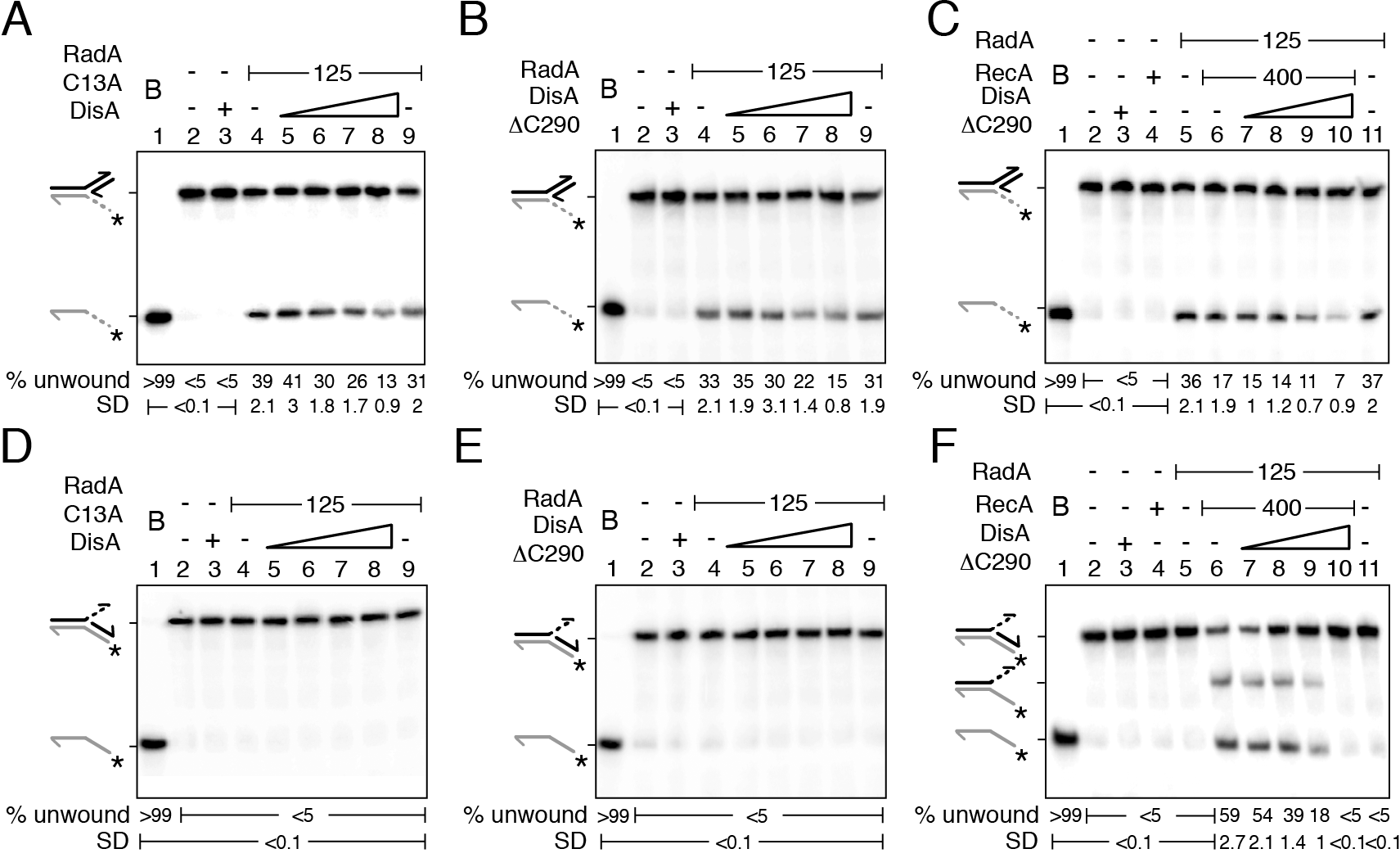


**Supplementary Figure S4.** DisA action on RadA helicase activity in the presence of RecA. (A-C) DisA or DisA ΔC290 partially inhibits RadA/Sms unwinding of 3´-fork DNA. (A) The DNA substrate was incubated with RadA/Sms C13A and increasing concentrations of DisA (100-800 nM). (B) The DNA was incubated with RadA/Sms (125 nM) and increasing concentrations of DisA ΔC290 (100-800 nM). (C) The DNA substrate was incubated with fixed concentrations of RadA/Sms and RecA and increasing concentrations of DisA ΔC290 (100-800 nM). (D-F) RecA loads RadA/Sms to unwind 5´-fork DNA. (D) The DNA was incubated with RadA/Sms C13A (125 nM) and increasing concentrations of DisA (100-800 nM). (E) The DNA substrate was incubated with RadA/Sms and increasing concentrations of DisA ΔC290 (100-800 nM). (F) The DNA substrate was incubated with fixed concentrations of RadA/Sms and RecA and increasing concentrations of DisA ΔC290 (100-800 nM). Reactions were done in buffer A containing 2 mM ATP (15 min, 30 ºC), and after deproteinization the substrate and products were separated by 6% PAGE and visualized by phosphor imaging. The quantification values of unwound DNA and the SD of >3 independent experiments are documented. Abbreviations: B, boiled DNA substrate; - and +, absence and presence of the indicated protein; * and grey color, the labelled strand; half of an arrowhead, the 3’ end.


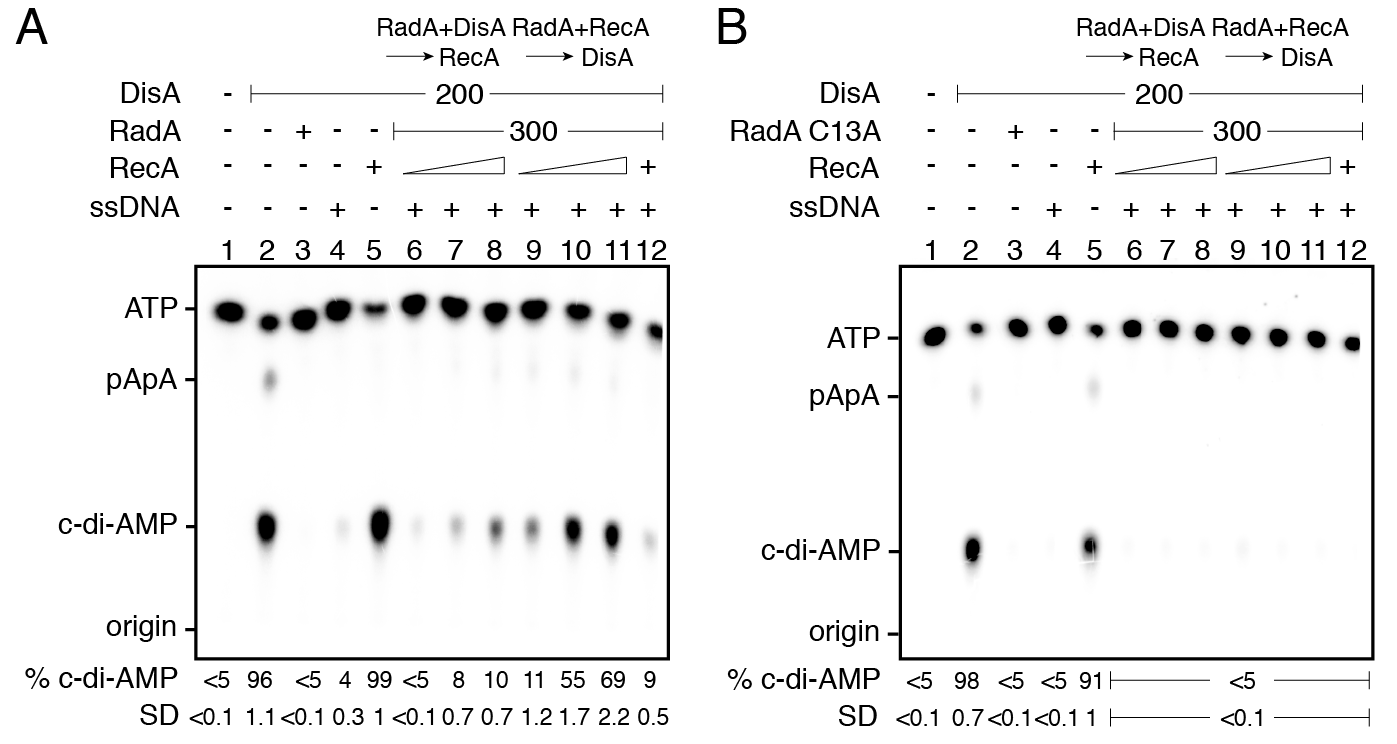


**Supplementary Figure S5.** RadA/Sms and ssDNA inhibit DisA DAC activity, but RecA counteracts this negative effect. DisA, or DisA and RadA/Sms (A) or RadA/Sms C13A (B), or DisA and RecA (1600 nM), or DisA and circular ssDNA (10 μM, in nt), or DisA, RecA (1600 nM), cssDNA (10 μM, in nt) and RadA/Sms (A) or RadA/Sms C13A were incubated in buffer C containing 100 μM [α^32^P]-ATP:ATP (30 min, 37 ºC). cssDNA and DisA (200 nM) were incubated with a fixed concentration of RadA/Sms (A) or RadA/Sms C13A (B) (300 nM) (5 min, 37 ºC), and then increasing RecA concentrations (400-1600 nM) were added in buffer C containing 100 μM [α^32^P]-ATP:ATP (30 min, 37 ºC). cssDNA, fixed RadA/Sms (A) or RadA/Sms C13A (B), and increasing RecA (400-1600 nM) concentrations were pre-incubated (5 min, 37 ºC), and then a fixed amount of DisA was added in buffer C containing 100 μM [α^32^P]-ATP:ATP (30 min, 37 ºC). The substrate, intermediates and products were separated by TLC and quantified. The quantification values of c-di-AMP synthesis and the SD of >3 independent experiments are documented. The position of [α^32^P]-ATP:ATP, linear pppA-pA (denoted as pApA), c-di-AMP and the origin are indicated.
